# Delivery routing problem of pure electric vehicle with multi-objective pick-up and delivery integration

**DOI:** 10.1371/journal.pone.0281131

**Published:** 2023-02-10

**Authors:** Wangang Cai, Yihao Zhang, Fuyou Huang, Chao Ma

**Affiliations:** 1 Hubei Key Laboratory of Power System Design and Test for Electrical Vehicle, Hubei University of Arts and Science, Xiangyang, China; 2 School of Automobile and Traffic Engineering, Hubei University of Arts and Sciences, Xiangyang, China; 3 Institute of Transportation Development Strategy & Planning of Sichuan Province, Chengdu, China; Guru Ghasidas Vishwavidyalaya: Guru Ghasidas University, INDIA

## Abstract

With the growth of people’s environmental awareness and the encouragement of government policies, the use of electric vehicles in logistics distribution is gradually increasing. In order to solve the dual demand of customers’ simultaneous pick-up and delivery in the “last kilometer logistics”, an electric vehicle routing problem with simultaneous pick-up and delivery and time window (EVRPSPDTW) is considered from the perspective of multi-objective distribution in this paper. Firstly, a decision-making model based on distribution cost and power consumption function is established. In this model, distribution cost includes transportation cost, vehicle use cost, penalty cost of not arriving on time and charging cost. Power consumption function is the energy loss caused by air resistance, tire rolling friction and transmission system. Secondly, a multi-objective genetic algorithm (NSGA-II) optimization solution with fast nondominated ranking and elite strategy is designed, and in view of the shortcomings of traditional NSGA-II, it is proposed to complete population initialization through greedy algorithm and random rules, introduce adaptive cross-mutation strategy in the chromosome crossing and mutation stage, and design three different neighborhood operators in mutation operation based on variant fitness function. Finally, the sensitivity analysis of traffic congestion coefficient further proves the effectiveness of the proposed model and the improved algorithm.

## 1 Introduction

With the development of society and the multiple needs of consumers, the concept of low carbon and the improvement of customer satisfaction have become the focus of logistics and distribution companies. Some data show that the transportation system is one of the main drivers of greenhouse gas emissions [1]. In practice, the fossil fuels that traditional internal combustion engine vehicles rely on are not only limited, but also cause serious environmental pollution. Therefore, major developed countries in the world have promoted new energy vehicles. As electric vehicles have no local greenhouse gas emissions and low noise environmental protection characteristics, the practice in recent years shows that electric vehicles will become an attractive low-carbon transportation in the road transportation system [2]. Although electric vehicles have the advantages of being friendly to the environment, the objective factors such as short mileage, long midway charging time and maximum load constraints can not be ignored. Electric vehicle routing problem has become another variant of vehicle routing problem (VRP). In this paper, the EV logistics distribution network is described as a directed graph in which the customer node, the charging pile in the distribution center, is taken as the node of the logistics transportation network, and the route between any two nodes is taken as the edge-to-edge route of the logistics transportation network, and the cost generated by the route is assigned as the weight of the edge of the logistics transportation network In recent years, the rapid development of optimization algorithms provides a basis for solving logistics distribution network models. Liu [3] proposed an improved ant colony optimization algorithm to solve the last mile problem of rural e-commerce logistics distribution, Li et al. [4] Applied a modified particle swarm optimization algorithm to the green vehicle routing problem of cold chain logistics considering greenhouse gas emissions, Ma et al. [5] and others developed an improved three-stage genetic algorithm based on Bertsimas robust discrete optimization theory in order to identify a highly robust and uncertain-insensitive electric vehicle (EV) assignment path.

In the VRP of green logistics, delivery time, pick-up and delivery have become a hot spot. In fact, the vehicle routing problem with simultaneous pick-up and delivery and time windows (VRPSDPTW) has not only time constraints, but also vehicle capacity constraints [6–8]. In addition, customer satisfaction with distribution services also affects the development of green logistics. In the previous research on distribution problems, the discussion of customer satisfaction mainly focused on logistics transportation with high time sensitivity, and it is usually the optimization of multiple objectives. Li et al. [9] used bilevel programming theory and genetic algorithm to solve the location routing optimization model of cold chain distribution center. Zhao et al. [10] solved the multi-objective cold chain distribution model based on the improved ant colony algorithm. Qin et al. [11] proposed on-time delivery as the standard to evaluate customer satisfaction, and used cyclic evolutionary genetic algorithm to solve the comprehensive cold chain vehicle routing optimization model. Yan et al. [12] incorporated customer satisfaction into the distribution problem with improved fuzzy delivery window, and further modeled the optimization problem as a multi-objective model with simultaneous delivery and pick-up. The above studies all considered customer satisfaction on the basis of low carbon, however, they did not discuss the particularity of electric vehicles. If the electric vehicle routing problem is extended to the vehicle routing problem with simultaneous pick-up and delivery, the influence of load change on electric vehicle power consumption should also be considered.

In practice, when planning the route of electric vehicles, it is necessary to calculate the energy required for driving and charge it when necessary, so as to manage its mileage. During the research on the energy consumption of electric vehicles, Xiao et al. [13] established a regression model of electric vehicle power consumption under the influence of driving speed and load. Shen et al. [14] proposed that the uncertainty of customer demand and the weight of electric vehicles are important factors affecting energy consumption. Pelletier et al. [15] and Bruni et al. [16] pointed out that weather and road information also have a certain impact on the energy consumption of electric vehicles. Therefore, in describing the dynamic circuit consumption of electric vehicles, the effects of driving distance, driving speed, vehicle load and traffic environment can be comprehensively considered.

As far as we know, the existing research on the routing problem of electric vehicle ignores the impact of changes in the traffic environment on the speed and arrival time of electric vehicles, thus affecting the power consumption of electric vehicle and the level of customer satisfaction during transportation. Because the electric vehicle is easily limited by the battery capacity and needs to be charged midway, some times the corresponding driving path needs to be changed. Therefore, it is very necessary for the path planning of the electric vehicle in the dynamic traffic environment. Nowadays, traffic congestion has become a very common phenomenon in the urban traffic network. Road congestion has an uncertain impact on the time and speed of vehicles traveling between the two nodes of the path. In the past, the vehicle routing problem considering traffic congestion was mainly focused on traditional fuel vehicle, and focused on single goals such as the lowest total cost of logistics distribution [17–19].

In reality, the logistics distribution of pure electric vehicle is affected by customer satisfaction, traffic congestion, simultaneous pick-up and delivery, specified time, and mileage. However, there is no study in the existing research that comprehensively takes these factors into account in the logistics path planning of electric vehicle. In summary, different from [11, 12], we consider VRP with simultaneous pick-up and delivery. Different from [6, 7], we discuss VRP with traffic congestion. Different from [17–19] concerning the route optimization of fuel vehicle, we focus on the route optimization of electric vehicle. Different from [13, 14] and [20], we take customer satisfaction into account and set VRP with multiple objective. Different from [8] and [21], we further investigate VRP with time window. The characteristics and contributions of our study are presented in Table 1.

**Table 1:** Summary of relevant literature.

Authors	Vehicle type		Time window		Objective Number		Customer satisfaction		Traffic jam		Simultaneous pick-up and delivery	
	Fuel vehicle	Electric vehicle	With	Without	Single	Multiple	With	Without	With	Without	With	Without
Qin et al. [11]	√		√			√	√			√		√
Yan et al. [12]	√		√			√	√			√		√
Zhou et al. [6]	√		√			√		√		√	√	
Zhou et al. [7]	√		√		√			√		√	√	
Zhu et al. [17]	√			√		√		√	√			√
Liu et al. [18]	√		√			√		√	√			√
Jie et al. [19]	√		√			√		√	√			√
Xiao et al. [13]		√		√		√		√		√		√
Shen et al. [14]		√		√		√		√		√		√
Goeke et al. [20]		√		√		√		√		√		√
Hornstra et al. [8]	√			√	√			√		√	√	
Basso et al. [21]		√		√	√			√		√		√
This paper		√	√			√	√		√		√	

Motivated by the research gap, in order to minimize the total logistics and distribution cost and maximize the average customer satisfaction, we reasonably plan the logistics and distribution route of pure electric vehicle with simultaneous pick-up and delivery and time windows under traffic congestion. We contribute to the literature in the following three aspects.

First, we consider battery energy consumption and traffic congestion, and establish a total logistics distribution cost model composed of charging cost, time window penalty cost, transportation cost and fixed cost. At the same time, we build a dynamic power consumption model under vehicle speed and dynamic load.

Second, we design a multi-objective genetic algorithm with fast non dominated sorting and elite reservation strategy (NSGA-II), and proposed an encoding and decoding method for the integrated electric vehicle routing problem of pickup and delivery, using both greedy algorithms and random rules. Different strategies are used to construct the initial population. Based on the classical genetic algorithm crossover mutation operator, an adaptive crossover y strategy is introduced, and three different neighborhood structures are designed in the population mutation stage. The effectiveness of the improved NSGA-II algorithm is proved by the comparison of the results of multiple simulation experiments. Finally, the sensitivity analysis of the traffic congestion coefficient is carried out in this paper, which further verifies the stability of the algorithm.

At last, we apply the modified example to discuss the optimal route distribution scheme when the algorithm gets the lowest total logistics distribution cost before and after the improvement. When only considering the lowest total cost of logistics and distribution, the total cost of logistics and distribution after the improved algorithm is reduced by 27.8%, while the average customer satisfaction is increased by 13.9%, and when only considering the highest average customer satisfaction, the total cost of logistics and distribution is reduced by 26.2%, and the highest average customer satisfaction level is the same as that of the NSGA-II algorithm before the improvement. Which proves that the algorithm designed in this paper can reasonably allocate vehicles to meet customers’ needs and reduce costs and increase efficiency when solving the logistics distribution route optimization problem of electric vehicles with simultaneous delivery. It provides some reference for enterprises in the actual logistics distribution process.

The remainder of this paper is structured as follows. Section 2 presents the assumptions and notations. Section 3 discusses the models. Section 4 proposes the solution algorithm. Section 5 analysis results. Section 6 concludes the paper.

## 2 Assumptions and notations

In order to minimize the total cost of logistics distribution and maximize the average customer satisfaction, this paper considers electric vehicle routing problem with simultaneous pick-up and delivery and time window (EVRPSDPTW). It aims to dispatch a team of electric logistics vehicles from a distribution center to provide logistics services for designated customers within a specified time window. Each customer has a positive supply demand and a reverse pick-up demand. The distribution vehicle starts from the distribution center and needs to load all the goods ready for distribution. When returning to the distribution center, it needs to take back all the recovered goods from customers. Both the pick-up and delivery must meet the vehicle’s own capacity limit. Due to electric vehicle power constraints, when necessary, the route needs to be planned for charging. When the remaining capacity of the battery is lower than the safe power threshold, it is necessary to find a suitable charging point for charging.

Specifically, the EVRPSDPTW problem can be modeled as a mixed integer linear programming model on a complete digraph *G*, where customers are modeled as graph vertices and paths between customers are modeled as graph arcs. The problem can be expressed as follows: *G* = (*V*_0,*N*+1_ ∪ *F*, *A*), in which *V*_0,*N*+1_ = *V* ∪ {0} ∪ {*N* + 1}(*V* = {1, …, *N*}) is a set of vertices, vertices 0 and *N* + 1 denote distribution centers, *V* represents a collection of customers. *F* represents the collection of charging points. *A* = {(*i*, *j*)|*i*, *j* ∈ *V*_0,*N*+1_, *F*, *i* ≠ *j*} is the set of arcs. The other related symbols and variables are summarized in Table 2.

**Table 2:** The notions and parameters.

Symbol	Description
$L_{i}^{k}$	The total path length accumulated by vehicle *k* when visiting customer *i*, where *i* ∈ *V* and *k* ∈ *K*
$sL_{i}^{k}$	The total path length of vehicle *k*, where *i* ∈ *V* and *k* ∈ *K*
$t_{i}^{k}$	The time when vehicle *k* arrives at customer node *i*, where *i* ∈ *V* and *k* ∈ *K*
$x_{0j}^{k}$	$x_{0j}^{k}=1$ indicates that vehicle *k* is used, otherwise $x_{0j}^{k}=0$, where *k* ∈ *K*, *j* ∈ *V*_*N*+1_∪*F*
$x_{ij}^{k}$	$x_{ij}^{k}=1$ indicates that vehicle *k* is charged at the charging point *j*, otherwise $x_{ij}^{k}=0$, where *k* ∈ *K*, *i* ∈ *V*_0_, *j* ∈ *F* and *i* ≠ *j*
$y_{N+1}^{k}$	$y_{N+1}^{k}=1$ means that vehicle *k* returns to the distribution center at last, otherwise $y_{N+1}^{k}=0$, where *k* ∈ *K*

$x_{ij}^{k}$ and $y_{N+1}^{k}$ are decision variables.

Based on the actual characteristics of logistics distribution and easy to abstract the problem into a model, the following assumptions are made:

1. The vehicles used by the logistics fleet are the same, with the same maximum load and battery capacity.
2. The departure time of the vehicle is the same, and it is fully charged when starting from the distribution center or leaving from the charging pile.
3. The acceleration process is ignored, and the electric vehicle runs at a constant speed in different periods of time.
4. The goods delivered and collected are the same type of products, and the loading and unloading process does not consume electricity.
5. Electric vehicles are transported and distributed on relatively flat roads.
6. The same charging station is allowed to be accessed many times and can be charged quickly within a certain period of time.
7. If the delivery vehicle arrives two hours later than the customer’s latest time window, it will be invalid.

## 3 The models

To better explain the multi-objective EVRPTWSPD problem, this section first analyzes the composition of distribution costs, and then establishes the models to minimize costs and maximize customer satisfaction.

### 3.1 Distribution cost analysis

The distribution costs of electric vehicle include fixed use cost of vehicle, transportation cost, charging cost and time window penalty cost.

Considering the fixed use cost of vehicle *C*_1_. It mainly includes vehicle start-up cost and labor cost. The operating cost per unit vehicle is *C*_*V*_, then

$$C_{1}=C_{V}\sum_{k\in K}\sum_{j\in V\cup F}x_{0j}^{k}.$$

Considering the transportation cost *C*_2_. There is a positive correlation between transportation distance and transportation cost. The vehicle purchase cost is *C*_*k*_, the maximum mileage is *Y*_*k*,max_, the distance between node *i* and node *j*(∀*j* ≠ *i*) is *d*_*ij*_, then

$$C_{2}=\sum_{k\in K}\sum_{i,j\in V\cup F}d_{ij}x_{{}_{i}j}^{k}c_{k}/Y_{k,\text{max}}.$$

Considering the charging cost. It is mainly related to the charging capacity of electric vehicle at the charging station. The higher the supplementary power at the charging station, the higher the charging cost. *C*_*e*_ denotes the unit electricity price, *b*_*i*_*j* denotes electric power consumption in unit distance of electric vehicle, then

$$C_{3}=\sum_{k\in K}\sum_{i\in V_{0}\cup F}\sum_{j\in V_{N}\cup F}c_{e}b_{ij}d_{ij}x_{ij}^{k}.$$

Considering the time window penalty cost *C*_4_. When the vehicle arrives early or late within the maximum time window acceptable to the customer, there will be a certain time waiting cost or penalty cost. The time window acceptable to the customer is [*ET*_*i*_, *LT*_*i*_], the waiting cost per unit time is *c*_*p*1_, the penalty cost per unit time is *c*_*p*2_, then

$$C_{4}=\sum_{k\in K}\sum_{i\in V}\left(c_{P1}max\left\{ET_{i}-t_{i}^{k},0\right\}+c_{p2}\;\text{max}\left\{t_{i}^{k}-LT_{i},0\right\}\right).$$

Analyze the energy consumption of electric vehicles, Goeke and Schneider [20], Basso [21] and others put forward that pure electric vehicles need to overcome rolling resistance *F*_*r*_, the vehicle’s own gravity *F*_*g*_ and gravity air resistance *F*_*a*_ of vehicles to do work without considering other resistance. Combined with the longitudinal dynamic equation of vehicles, we can get the following formula:

$$F_{g}=mg\;\text{sin}\theta \left(t\right).$$

$$F_{r}=mgf\;\text{cos}\theta \left(t\right).$$

$$F_{a}=0.5RA\rho v_{t}^{2}.$$

From the above formula, it can be deduced that the mechanical power *P*_*M*_ of electric vehicle at constant speed is:

$$\begin{array}{c} P_{M}\left(t\right)=F_{g}+F_{r}+F_{a}=mg\text{sin}\theta \left(t\right)+mgf\text{cos}\theta \left(t\right)+0.5RA\rho v_{t}^{2}. \end{array}$$ (1)

When the electric vehicle runs at a constant speed, we also need to consider the factor of battery energy loss in the power consumption model. When the mechanical power of the electric vehicle is positive when the power system of the electric vehicle is in traction mode, we will get the conversion relationship between mechanical power PME power $P_{E}^{d}$ and battery power $P_{B}^{d}$ [22].

$$P_{E}^{d}=\phi^{d}P_{M},P_{M}\geq 0.$$

$$P_{B}^{d}=\varphi^{d}P_{E}^{d},P_{E}^{d}\geq 0.$$

By sorting out the above formulas, the power consumption model of electric vehicles at a constant speed for a period of time can be obtained as follows:

$$\begin{array}{c} b_{ij}=\phi^{d}\varphi^{d}\left(\frac{\left[g\text{sin}\theta \left(t\right)+gf\text{cos}\theta \left(t\right)\right]\left(G+w_{ij}\right)}{3600}+\frac{RA\rho v_{t}^{2}}{76140}\right)v_{t}t_{ij}. \end{array}$$ (2)

From the power consumption model, it can be seen that the dynamic load changes of EV after loading and unloading, and the speed changes of EV caused by traffic environment will have an uncertain impact on the power consumption of EV and then affect its timeliness. The symbols designed in the above formula have the following meanings: where *φ*^*d*^ is the output efficiency parameter representing the work done by the driving motor, *φ*^*d*^ is the output efficiency parameter of the battery, *f* is the rolling resistance coefficient, *R* is the air resistance coefficient,*ρ* is the density of air. *θ*(*t*) is the road slope at this time, then the average slope value will be used instead due to it is difficult to measure the road slope in real time. *G* is the weight of the vehicle, *w*_*ij*_ is vehicle load, *A* is the windward area of the vehicle, *t*_*ij*_ is the time when the car runs at a constant speed during this period, *v*_*t*_ is the speed of the vehicle in this period.

It is worth noting that the speed of the vehicle will also change due to the influence of the traffic environment in different periods. Therefore, we establish the speed equation under traffic congestion.

$$\begin{array}{c} v_{t}=\frac{v_{f}}{\rho_{t}}, \end{array}$$ (3)

where *v*_*f*_ is the speed at which the car is moving smoothly, *ρ*_*t*_ is the traffic congestion coefficient.

### 3.2 Mathematical models

Firstly, we consider the problem of minimizing the total cost of logistics distribution, which has the following models.

$$\begin{array}{c} \text{min}\;Z=\sum_{k\in K}\left(C_{1}+C_{2}+C_{3}+C_{4}\right). \end{array}$$ (4)

s.t.

$$\begin{array}{c} \sum_{k\in K}\sum_{j\in V_{N+1}\cup F}x_{ij}^{k}=1,\;i\in V,i\neq j. \end{array}$$ (5)

$$\begin{array}{c} \sum_{k\in K}\sum_{j\in V_{N+1}\cup F}x_{ij}^{k}\leq 1,\;i\in F,i\neq j. \end{array}$$ (6)

$$\begin{array}{c} \sum_{k\in K}\sum_{i\in V_{N+1}\cup F}x_{ji}^{k}-\sum_{k\in K}\sum_{i\in V_{0}\cup F}x_{ij}^{k}=0,\;i\neq j,j\in V\cup F. \end{array}$$ (7)

$$\begin{array}{c} \sum_{k\in K}\sum_{j\in V\cup F}x_{0j}^{k}+\sum_{k\in K}\sum_{j\in V\cup F}x_{jN+1}^{k}=2y_{N+1}^{k}. \end{array}$$ (8)

$$\begin{array}{c} t_{i}^{k}+\tau_{w}^{i}+\left(t_{ij}+s_{i}\right)x_{ij}^{k}-l_{0}\cdot \left(1-x_{ij}^{k}\right)\leq t_{j}^{k},\forall i\in V_{0},\forall j\in V_{N+1}\cup F,i\neq j. \end{array}$$ (9)

$$\begin{array}{c} \tau_{j}=\tau_{i}+\tau_{w}^{i}+\left(t_{ij}^{k}+s_{i}\right)x_{ij}^{k},\forall i\in V_{0},\forall j\in V_{N+1}\cup F,i\neq j. \end{array}$$ (10)

$$\begin{array}{c} \tau_{w}^{i}=\text{max}\left\{0,\left[ET_{i}-t_{{}_{i}}^{k}\right]\right\}. \end{array}$$ (11)

$$\begin{array}{c} z_{0}=C,y_{0}=Q. \end{array}$$ (12)

$$\begin{array}{c} 0\leq z_{j}\leq z_{i}-x_{ij}^{k}\left(p_{i}-q_{i}+C\right)+C,\forall i\in V_{0}\cup F,\forall j\in V_{N+1}\cup F,i\neq j. \end{array}$$ (13)

$$\begin{array}{c} 0\leq y_{j}\leq y_{i}-x_{ij}^{k}\left(b_{ij}+Q\right)+Q,\forall j\in V_{N+1}\cup F,\forall i\in V,i\neq j. \end{array}$$ (14)

$$\begin{array}{c} 0\leq y_{j}\leq Q-b_{ij}x_{ij}^{k},\forall j\in V_{N+1}\cup F,\forall i\in 0\cup F,i\neq j. \end{array}$$ (15)

$$\begin{array}{c} {\text{sL}}_{i}^{k}\geq L_{i}^{k}+d_{i0}x_{i0}^{k},\forall i\in V,k\in K. \end{array}$$ (16)

$$\begin{array}{c} {\text{sL}}_{i}^{k}\leq L_{i}^{k}+d_{i0}-Lx_{i0}^{k}+L\sum_{j\in V}x_{0j}^{k}\forall i\in V,k\in K. \end{array}$$ (17)

In the above-mentioned mathematical models, Eq (4) represents the minimization of the total transportation cost. Formulas (5), (6) and (7) guarantee the connection between arcs and flow conservation, and each customer is only visited once. Eq (8) indicates that each vehicle must return to the center from the distribution center.

Formulas (9) and (10) ensure the rationality of the travel time and time window of electric vehicles between nodes, in which $\tau_{w}^{i}$ indicates the waiting time that may occur, *s*_*i*_ is the time of serving customers or charging time at the charging pile.

Eq (11) represents the time range of waiting for customer service. Formulas (12), (13), (14) and (15) ensure that the load and remaining battery capacity of the electric vehicle in arc (*i*, *j*) do not exceed the vehicle maximum load and battery capacity constraints, where *C* and *Q* respectively represent the maximum load of the vehicle and the rated capacity of the battery, *z*_*j*_ and *y*_*j*_ represent the residual load capacity and the remaining battery capacity.

Formulas (16) and (17) indicate that in order to avoid driver fatigue driving, the maximum driving path distance of the vehicle shall not exceed *L*.

Secondly, we consider the problem of maximizing average customer satisfaction, we have the following model.

$$\begin{array}{c} \text{max}\;F=\frac{1}{n}\sum_{i=1}^{n}s_{i}t_{i}. \end{array}$$ (18)

For logistics enterprises, customer satisfaction reflects the service level of enterprises. In the traditional time window, vehicles arriving early and late are treated equally, which obviously does not meet the psychological expectations of customers. When serving customers outside the expected but acceptable time window, although it is allowed by customers, the level of customer satisfaction will decline to a certain extent [23]. Therefore, this paper refers to the method of fuzzy time window [24] to reflect the level of customer satisfaction.

By supposing that the acceptable time window range for customer *i* is [*ET*_*i*_, *EL*_*i*_], the ideal time window range is [*A*_*i*_, *B*_*i*_], the time sensitivity coefficient is *β*(> 0). Then the membership function of customer *i*’s satisfaction is calculated as follows:

$$\begin{array}{c} s_{i}\left(t_{i}\right)=\{\begin{array}{cc} {\left(\frac{t_{i,k}-ET_{i}}{A_{i}-ET_{i}}\right)}^{\beta }, & t_{i}^{k}\in \left[ET_{i},A_{i}\right],\forall i\in V\cup F,k\in K,\\ 1, & t_{i}^{k}\in \left[A_{i},B_{i}\right],\forall i\in V\cup F,k\in K,\\ {\left(\frac{LT_{i}-t_{i,k}}{LT_{i}-B_{i}}\right)}^{\beta }, & t_{i}^{k}\in \left[B_{i},LT_{i}\right],\forall i\in V\cup F,k\in K,\\ 0, & t_{i}^{k}\notin \left[ET_{i},LT_{i}\right],\forall i\in V\cup F,k\in K. \end{array} \end{array}$$ (19)

In summary, the multi-objective optimization model of pure electric vehicle logistics and distribution route planning with integrated pick-up and delivery with time window is as follows:

$$\begin{array}{c} \left\{ \begin{array}{c} \text{min}\;F=min\{Z,1-F\}\\ s.t.\;Equations\;from\;Eq.(5)\;to\;Eq.(17)\;hold. \end{array}\right. \end{array}$$ (20)

## 4 Solution algorithm

**Fig 1:**
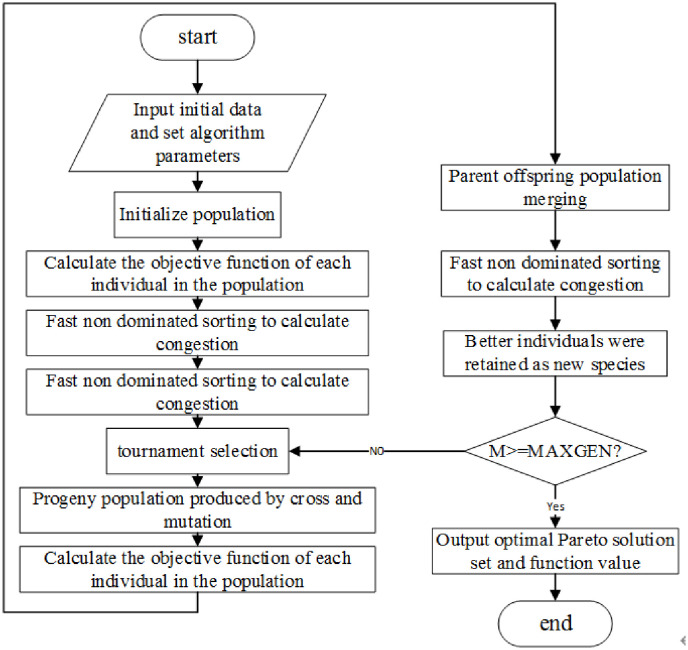
Flow chart of multi objective optimization algorithm based on NSGA-II.

The classical multi-objective vehicle routing problem (MO-VRP) mainly focuses on the traditional fuel vehicles. The multi-objective EVRPSDPTW proposed in this paper is another extension of the classical MO-VRP. The problem studied in this paper has two optimization objectives, it is hardly to achieve all the optimal objectives at the same time through a single solution. The crossover and mutation operators of genetic algorithm are especially suitable for solving multi-objective optimization problems. Therefore, this paper proposes a fast non dominated sorting genetic algorithm (NSGA-II) with elite strategy to obtain a set of Pareto optimal solutions. The flow of the multi-objective optimization algorithm based on NSGA-II is shown in Fig 1:

### 4.1 Solution coding

Before initializing the chromosome population, we first need to encode the chromosomes. First of all, to meet our basic requirements when modeling, that is, each customer can only be served by one vehicle, so this paper uses natural number coding [25] to solve the problem of electric vehicle path planning. In order to ensure sufficient redundancy of chromosomes, we set the staining length to 2N+M+1, where N is the number of customers, and M is the number of targets.

### 4.2 Initialize the population

In order to improve the quality of the initial population and maintain the diversity of the population, we construct a mixed initial population in two ways.

1. The pre-NP-1 individuals in the population randomly generated N-length chromosome fragments through the Randperm function built in MATLAB, and NP represented the number of the population.
2. The last individual in the population is constructed with the help of a greedy algorithm, The specific method is as follows:
  Step 1: Establish a distance matrix of customers with N rows and N columns, set the visited customer set as *C*, and the initial set as an empty set. The set of unvisited customers is *U* = {1, 2, …, *N*}, where N is the number of customers.
  Step 2: Assign all the zeros on the diagonal of the distance matrix to infinity. Find out the row number and column number corresponding to the minimum distance from the distance matrix. If there are multiple minimum distances, select the first number in the row number set as the starting point of the initial route.
  Step 3: Add the visited customer to the visited customer set *C*, delete the customer in the set *U*, complete the update, and assign the starting customer as the immediately preceding point as pre-point.
  Step 4: Find the distance between the pre-point and the city in the corresponding row in the distance matrix, which is denoted as pre-dist. Set all the columns corresponding to the visited cities in the pre-dist to infinity, find the column ordinal number corresponding to the smallest value in the pre-dist as the next pre-point, and update the sets *C* and *U*.
  Step 5: If all cities have been accessed, set *C* is the initial route for the final construction. Otherwise proceed to steps 3 and 4.
3. After each completion of the construction of the initial chromosome fragment, we need to ensure the validity of the chromosome, that is, each customer node on the chromosome should not exceed the maximum load of the vehicle after completing delivery and pickup, and insert the distribution center “1” at the critical point that meets the maximum load constraint of the vehicle to form the service route of each vehicle. And so on until all customers have been assigned, forming our initial delivery route. After simple adjustments, the initial distribution path is converted to individuals in the initial population.
4. It is worth noting that the coding process of chromosomes does not include charging piles, to avoid charging piles participating in crossover and mutation operations, and to decode the individuals in the population after completing the operation of population renewal and then insert them into the charging pile.

### 4.3 Fitness assessment

When evaluating the fitness of individuals in the population, we need to calculate the objective function value of our optimization problem.

First of all, we need decode the individuals in the initial population. At this point, we need to insert the charge point in the decoded initial distribution path. We insert the charge point based on the following three conditions. The first one is that it is necessary to consider looking for the nearest charging point for charging if the power of the car at a certain point on the path is less than 30% of the remaining power. The second one is that it needs to find the nearest charge point at the previous customer node for charging if the remaining power at this point cannot reach the nearest charge point. The last one is that all power consumption calculations are based on the dynamic power consumption model proposed in Eqs (2) and (3).

After completing the operation in the previous step, after obtaining the initial distribution scheme including charging piles, in addition to calculating the total logistics distribution cost of the initial distribution scheme and the average customer satisfaction, we also need to judge whether the current scheme meets the constraints of customer time window, load, remaining power and maximum path length, and record the times of violating the constraints.

In sum, NSGA-II algorithm generally takes the minimum target as the best, so the fitness function is established as follows:

$$\begin{array}{c} fit=Z-F+overrestrian*G, \end{array}$$ (21)

where *Z* is the total cost of logistics distribution. *F* is customer satisfaction, means that the load, latest time and other constraints are not meet. *G* can be regarded as an infinite penalty weight.

### 4.4 Fast non-dominated sorting

Before performing the population update operation, it is necessary to sort the Pareto solution set at a non-dominated level. We compare the solutions in the population pairwise. Each individual *p* in the population is assigned two parameters *S*_*i*_ and *np*, *S*_*i*_ represent the set of solutions dominated by the solutions of individual *p*, and *np* represents the number of dominating individual *p*. The specific steps are as follows:

Step1: Set the parameters of the first individual *p* in the population, *np* = 0 and *S*_*i*_ = Φ;
Step2: Compare the solution of individual *p* with the solution of other individuals *q*(*q* ≥ 2) in the population for non-dominance relations, turn to step 3;
Step 3: Non-dominance relationship comparison. If *p* and *q* are two feasible solutions to minimize the multi-objective optimization problem, if conditions (i) and (ii) are met, then solution *p* is considered to be superior to solution *q*, that is, solution *p* dominates solution *q*, *m* represents the number of objective functions;
  (i) ∀*m* ∈ {1, 2, …, *k*}, *f*_*m*_(*p*) ≤ *f*_*m*_(*q*);
  (ii) ∃*m* ∈ {1, 2, …, *k*}, *f*_*m*_(*p*) > *f*_*m*_(*q*).
Step4: In each comparison, if the solution *p* dominates the solution *q*, *q* is counted in the set *S*_*i*_ of the solutions dominated by *p*, and if the solution *q* dominates the solution *p*, the number of solutions that dominate *p* is increased by 1. The solution of *np* = 0 is the non-dominated solution, and the set of solutions that find all *np* = 0 is denoted as *F*_1_, which is the first non-dominated level.
Step5: Calculate every solution *p* in the previous non-dominant hierarchy, and the set of solutions dominated by solution *p* is *S*_*i*_. Then subtract 1 from the parameter *np* of all solutions *q* in *S*_*i*_ to find out that the set of solutions with parameter *np* equal to 0 is the next non-dominant hierarchy, and so on until all the non-inferior solution sets are layered.

### 4.5 Population regeneration

(1) Selection. In this paper, the binary tournament is used to select the better individual. By selecting two individuals from the parent population each time and comparing their non-dominated hierarchy ranking and crowding degree [26], individuals with better non dominated hierarchy or the same non-dominated hierarchy but higher crowding degree are selected until the preset number of offspring population is met, and then genetic operator operation is performed.

(2) Crossover and mutation. In this paper, the position-based crossover (PBX) [27] and the use of different neighborhood operators are used to crossover individuals in the offspring population selected through the tournament and gene mutation operations, which can improve the diversity of the population. At the same time, the local search ability of the algorithm is enhanced. The crossover mutation probability is determined by the adaptive crossover mutation operator. The calculation formula is as follows:

$$P_{c}=\{\begin{array}{c} \frac{p_{c}\left(\text{min}\left\{f\left(i_{1}\right),f\left(i_{2}\right)\right\}-f_{\text{min}}\right)}{f_{avg}-f_{\text{min}}},\text{min}\left\{f\left(i_{1}\right),f\left(i_{2}\right)\right\}\geq f_{avg},\\ p_{c},\text{min}\left\{f\left(i_{1}\right),f\left(i_{2}\right)\right\}<f_{avg}. \end{array}$$

$$P_{m}=\{\begin{array}{c} \frac{p_{m}\left[f\left(i\right)-f_{\text{min}}\right]}{f_{avg}-f_{\text{min}}},f\left(i\right)\geq f_{avg},\\ p_{m},f\left(i\right)<f_{avg}. \end{array}$$

*P*_*m*_ is the crossover fitness function, *p*_*m*_ is the crossover probability. *P*_*c*_ is the mutation fitness function and *p*_*c*_ is the mutation probability. *f*(*i*_1_) and *f*(*i*_2_) are the fitness values of the individual to be crossed, *f*(*i*) is the fitness value of the individual to be mutated at present. *f*_min_, *f*_*avg*_ is the minimum fitness value and the average fitness value of the current population. From the fitness function formula, it can be seen that the smaller the fitness value is, the higher the quality of the individual. Therefore, we should carry out crossover mutation operation on the individual whose fitness value is higher than the average fitness value of the population with greater crossover mutation probability to avoid the population falling into local optimal solution and improve the population quality.

(3) Neighborhood operation. In mutation operation, we design three different neighborhood operators of exchange, reversal and insertion, and select different neighborhood structures by roulette function according to the probability to execute the corresponding gene operation. The probability of selecting exchange structure is pSwap = 0. 2, the probability of selecting reversal structure is pReversion = 0. 5, and the probability of selecting insertion structure is pInsertion = 0. 3.

Exchange operation: We randomly select two genes at different positions on the current chromosome to exchange elements at these two positions.

Reverse operation: Randomly select genes at two different positions on the current chromosome and then arrange the elements between these two positions in reverse order.

Insertion operation: Randomly select two genes at different positions on the current chromosome and insert the element at the first position after the second element.

**Fig 2:**
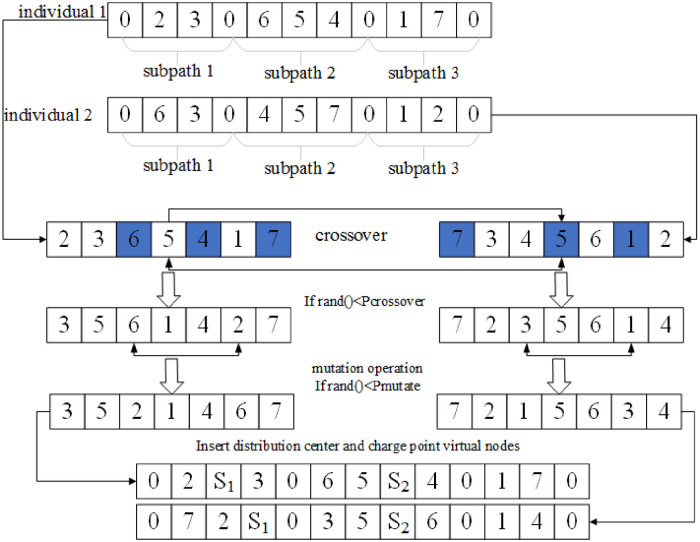
Path individual genetic operation with charging point nodes.

Therefore, this paper updates the population through the path individual genetic operation process with charging point nodes as shown in Fig 2.

### 4.6 Elite retention strategies

**Fig 3:**
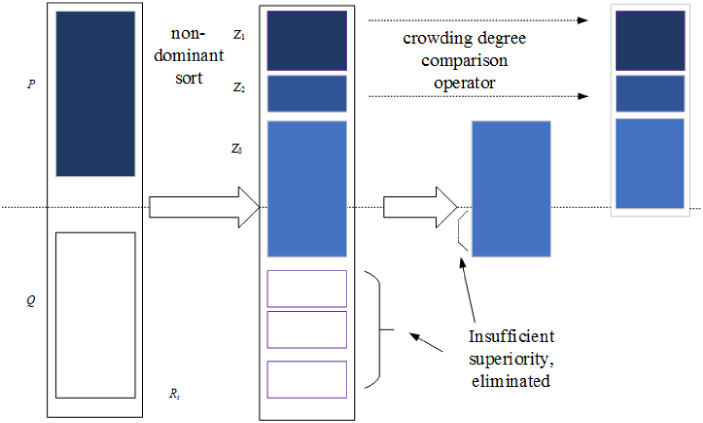
Elite retention process.

Refer to the research of Deb and Goel [28] (as shown in Fig 3). Elite retention means ensuring that the best individuals of the previous generation enter the next generation. In this paper, the solutions in Pareto solution set are sorted from small to large according to the non dominated hierarchy, In the non-dominated hierarchy. The solutions are sorted from large to small by comparing the degree of crowding, so that the solutions in the solution set can be sorted from good to bad. Then retain the top *N* individuals with the best sorting (*N* is the number of offspring populations) as the next generation parent individuals.

## 5 Results and discussion

**Fig 4:**
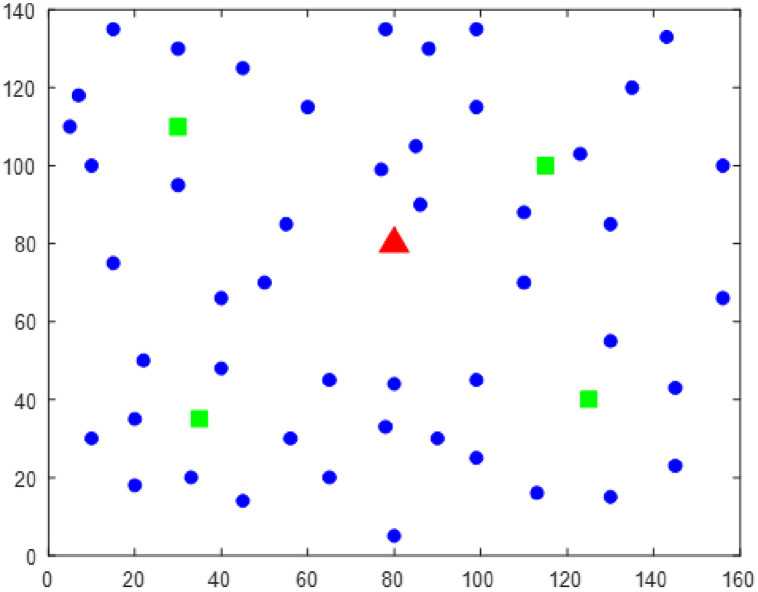
Location map of logistics distribution system.

In this paper, the time range of customer service is calculated according to the customer’s demand. The example includes 48 customers, 1 distribution center and 4 charging piles, The logistics distribution service information and charging pile location of some customers are shown in Table 3. The number 49–52 represents the charging station, and the specific distribution system is shown in Fig 4. In order to make the research closer to reality, this paper considers the impact of traffic congestion on the logistics transportation process. According to the traffic situation of the morning and evening peak in the city, We take the two time periods of 7:00–9:00 a.m. and 17:00–19:00 p.m. as the time period of traffic congestion, and drive normally in the rest of the time, The departure time of the logistics fleet is set at 7 a.m. According to the characteristics of electric vehicles, the electric vehicles drive at a constant speed of 60km/h under normal conditions and 30km/h in congestion. The relevant energy consumption calculation parameters are shown in Table 4, and other cost calculation parameters are shown in Table 5. In order to get better simulation results, the parameters of NSGA-II algorithm are set as follows: the number of population is 80, the maximum number of iterations is 500, the crossover probability is 0.8, and the mutation probability is 0.2.

**Table 3:** Logistics distribution information and parameters.

Node	Coordinate	Maximum time window	Ideal time window	Volume of demand (kg)	Quantity of pick up (kg)	Service time (h)
0	(80,80)	(0:00–24:00)	(0:00–24:00)	0	0	0
1	(40,66)	(8:00–10:20)	(8:40–9:40)	150	130	0.3
2	(56,30)	(11:00–13:00)	(11:40–12:20)	255	125	0.4
3	(99,45)	(9:00–11:00)	(9:20–10:30)	80	90	0.2
4	(110,70)	(8:20–10:35)	(8:30–10:00)	86	32	0.2
5	(10,30)	(9:00–11:30)	(9:30–11:00)	78	69	0.15
6	(40,48)	(14:00–16:35)	(14:20–16:00)	190	120	0.4
7	(30,95)	(7:00–9:00)	(7:20–8:20)	266	140	0.6
8	(78,33)	(7:30–9:15)	(7:50–9:40)	110	80	0.3
9	(130,135)	(8:20–10:45)	(8:40–10:10)	60	50	0.15
10	(30,130)	(14:20–16:00)	(14:40–15:20)	40	20	0.1
49	(30,110)	(0:00–24:00)	(0:00–24:00)	0	0	0.5
50	(35,35)	(0:00–24:00)	(0:00–24:00)	0	0	0.5
51	(115,110)	(0:00–24:00)	(0:00–24:00)	0	0	0.5
52	(125,40)	(0:00–24:00)	(0:00–24:00)	0	0	0.5

**Table 4:** EV related energy consumption calculation parameters.

Symbol	Meaning	Value
*G*	Vehicle weight	3760 kg
*C*	Battery capacity	45 kWh
*g*	Acceleration of gravity	9.8 m/s^2^
*R*	Air drag coefficient	0.7
*f*	Rolling resistance coefficient	0.012
*ρ*	Air density	1.2041
*Q*	Rated load	1335
*A*	Windward area of vehicle	3.8 M^2^
*ϕ* ^*d*^	Motor output efficiency parameters	1.184692
*φ* ^*d*^	Battery output efficiency parameters	1.112434

**Table 5:** Cost parameters related to distribution process.

Parameter	Value
Unit use cost of vehicle	200 RMB per vehicle
Vehicle purchase cost	250 thousand RMB
Scrapped mileage	600 thousand km
Early arrival penalty cost	10 RMB per hour
Late arrival penalty cost	40 RMB per hour
Average unit charging cost	0.7 RMB per kWh
Time sensitive coefficient *β*	0.8
Maximum path length	400 km

According to the experimental examples and models in the traffic congestion environment, we repeated the multi-objective genetic algorithm before the improvement and the improved multi-objective genetic algorithm (H-NSGA-II) for 10 simulation experiments. Each time NSGA-II algorithm will generate a set of Pareto solutions, and the Pareto solution front is the optimal solution set obtained by NSGA-II algorithm in each run, The solutions in the solution set are mutually non-dominant. The characteristics of the multi-objective optimization algorithm determine that the solutions generated by the operation cannot obtain all the optimal values. The experimental data generated by each operation are shown in Table 6. *Z*_*i*_ represents the lowest total logistics distribution cost in Pareto solution, *F*_*i*_ represents the average customer satisfaction when obtaining the lowest total logistics distribution cost, *F*_*j*_ represents the highest average customer satisfaction in Pareto solution, and *Z*_*j*_ represents the total logistics distribution cost when obtaining the highest average customer satisfaction.

**Table 6:** Algorithm running results comparison.

H-NSGA-II					NSGA-II				
Number of runs	*Z*_*i*_	*F*_*i*_	*F*_*j*_	*Z*_*j*_	Number of runs	$Z_{i}^{\prime }$	$F_{i}^{\prime }$	$F_{j}^{\prime }$	$Z_{j}^{\prime }$
1	3175.527	0.603	0.733	3528.474	1	4355.570	0.570	0.691	4613.455
2	2866.601	0.595	0.733	3320.159	2	4732.341	0.527	0.714	5020.065
3	2937.497	0.691	0.709	3013.925	3	4436.642	0.547	0.776	4928.952
4	3067.443	0.667	0.750	3260.790	4	4441.981	0.641	0.687	4606.592
5	3155.708	0.645	0.726	3926.615	5	4211.472	0.635	0.722	4412.002
6	3125.728	0.584	0.742	3324.600	6	3933.184	0.588	0.656	4632.258
7	3423.291	0.643	0.720	3580.356	7	4334.443	0.583	0.717	4811.160
8	3269.088	0.590	0.726	3665.436	8	4308.958	0.594	0.711	4514.016
9	3426.951	0.685	0.704	3472.928	9	4379.646	0.626	0.716	4624.648
10	2807.701	0.667	0.715	3466.827	10	4077.538	0.595	0.690	4236.418

In order to prove the superiority of the improved multi-objective genetic algorithm (H-NSGA-II) proposed in this paper, the t-test significance difference method is used to test it based on matlab platform. Because the population mean and variance are unknown, the lili-test function is used to test the normal distribution characteristics of each group of sample data, and the following assumptions are made:

H0: indicates that the sample obeys normal distribution.
H1: indicates that the sample does not obey normal distribution.

When the confidence level is 0.05, the original hypothesis is accepted when *h* = 0, *p* ≥ 0.05, and the original hypothesis is rejected when *h* = 1, *p* < 0.05 from the test results in Table 7, it can be seen that each group of data obeys normal distribution and can be tested by t-test significance difference.

**Table 7:** Lilie-test analysis results.

		H-NSGA-II				NSGA-II			
Sample size		10				10			
Objective function value		*Z*_*i*_	*F*_*i*_	*F*_*j*_	*Z*_*j*_	$Z_{i}^{\prime }$	$F_{i}^{\prime }$	$F_{j}^{\prime }$	$Z_{j}^{\prime }$
Normal parameter	Average Standard deviation	3125.554	0.637	0.726	3456.011	4327.209	0.591	0.708	4680.835
		202.525	0.039	0.014	235.737	205.874	0.035	0.030	221.379
*h*		0	0	0	0	0	0	0	0
*p*		0.50	0.32	0.50	0.50	0.38	0.50	0.15	0.22

After verifying the normal distribution characteristics of sample data, t-test test is carried out to make assumptions for each group of data:

H0: The mean difference of the same type of objective function values under the two algorithms is 0.
H1: The mean difference of the same type of objective function values under the two algorithms is not 0.

Set the confidence level to 0.05. When *h* = 1 and *p* < 0.05, it means that the null hypothesis H0 does not hold and the difference is significant, otherwise the H0 hypothesis is accepted. Through the t-test test on the four pairs of sample data, the corresponding test results are shown in Table 8.

**Table 8:** t-test analysis results.

	$\left(Z_{i},Z_{i}^{\prime }\right)$	$\left(F_{i},F_{i}^{\prime }\right)$	$\left(F_{j},F_{j}^{\prime }\right)$	$\left(Z_{j},Z_{j}^{\prime }\right)$
*h*	1	1	0	1
*p*	8.4579*e*^−7^	0.0095	0.2124	1.0214*e*^−5^

From Table 8, it can be seen that the data between the lowest total logistics distribution cost, the lowest average customer satisfaction, and the highest total customer distribution cost are analyzed by t-test and accept the hypothesis of H1. That is, the lowest total logistics distribution cost before and after the algorithm improvement, the lowest The mean difference between the average customer satisfaction and the highest total cost of logistics and distribution is significant, and the data analysis between the highest average customer satisfaction accepts the assumption proposed by H0. That is, there is no significant difference in the mean of the highest average customer satisfaction.

By comparing the mean values between them, it can be concluded that the lowest total logistics distribution cost obtained by the improved algorithm is reduced by 27.8%, while the average customer satisfaction is increased by 13.9%. While the highest average customer satisfaction remained basically unchanged, the highest logistics and distribution costs decreased by 26.2 percent. In addition, the average running time of the algorithm before the improvement is about 88s, and the average running time of the improved algorithm is about 99s, and the running time of the algorithm is within a reasonable range.

**Fig 5:**
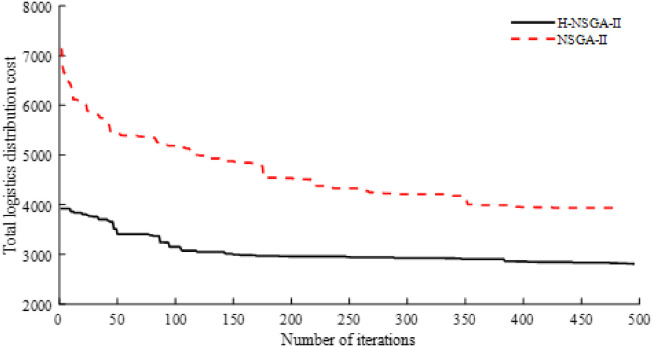
Comparison of convergence results of algorithms.

In order to compare the difference of convergence before and after the improvement of the algorithm, this paper selects the optimal solution with the lowest total logistics distribution cost in 10 simulation experiments to compare the iterative convergence curves, as shown in Fig 5.

From the above analysis, it can be seen that the improved algorithm in this paper has better performance than the traditional NSGA-II in solving the problem of multi-objective simultaneous delivery electric vehicle logistics distribution considering traffic congestion It can be seen from Fig 5 that the improved NSGA-II algorithm converges in the 496th generation and the traditional NSGA-II algorithm converges in the 426th generation The improved NSGA-II algorithm can find the sub-optimal solution with high probability in the early and middle stages with strong searching ability, while the traditional NSGA-II algorithm has fallen into the local optimum and can’t jump out in the middle and late stages.

In-depth analysis in the traditional NSGA-II algorithm, the initial solution is constructed completely by random rules, on the one hand, the population quality cannot be guaranteed, on the other hand, the optimization time of the algorithm is too long and it is easy to fall into local optimum The hybrid NSGA-II algorithm designed in this paper introduces greedy algorithm and random rules in the initial population construction stage, which can keep the diversity of the population and ensure the quality of the population, which is beneficial to accelerate the convergence speed of the algorithm In order to avoid falling into local optimum, this paper introduces adaptive crossover mutation strategy in the crossover mutation stage, which makes the individuals whose quality is lower than the average level carry out crossover mutation operation with greater probability, and designs three different neighborhood operations in the mutation stage, which improves the disturbance mechanism of the algorithm, reduces the possibility of falling into local optimum and further increases the diversity of the population. The elite retention strategy in NSGA-II retains the excellent individuals in the iteration process, so the three improved strategies proposed in this paper can make the population evolve in a better direction and finally make the algorithm converge to a better suboptimal solution.

**Fig 6:**
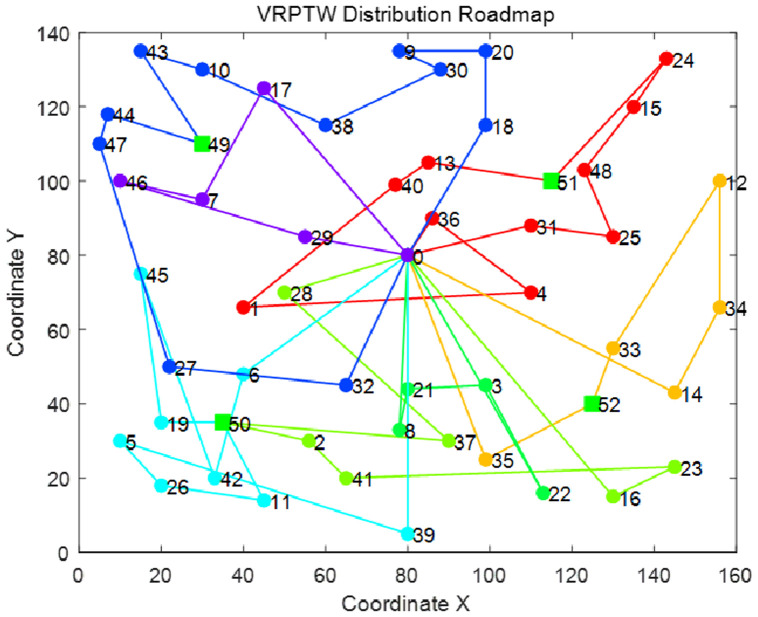
H-NSGA-II optimal path.

**Fig 7:**
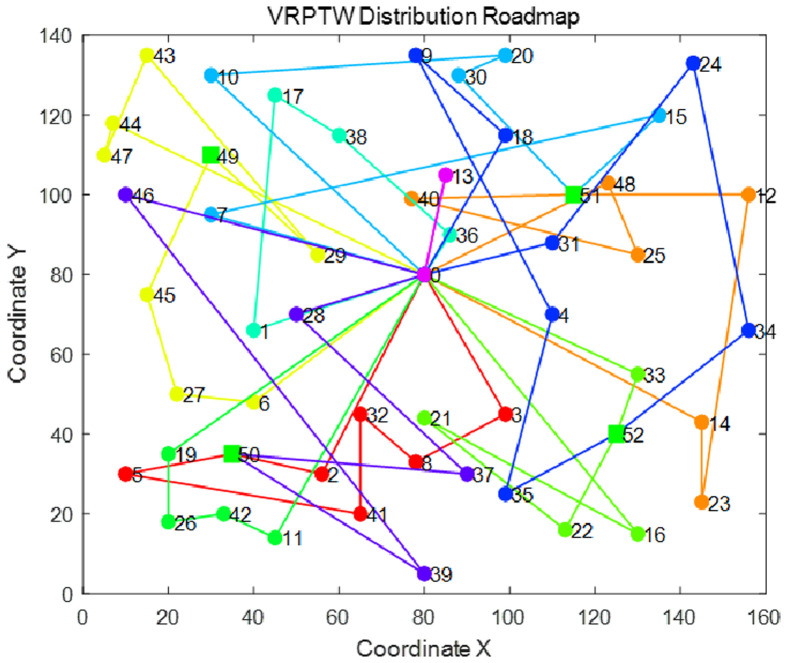
NSGA-II optimal path.

In order to show more intuitively the effect of the algorithm to solve the vehicle routing problem of electric vehicles taking and delivering goods at the same time, the route planning diagram of the minimum total cost of logistics distribution before and after the improvement of NSGA-II algorithm in 10 experiments is shown in Figs 6 and 7, and the specific route is shown in Tables 9 and 10. Where VN represents the vehicle number VR represents the vehicle route VAT represents the arrival time of the vehicle/represents the arrival of the charging pile and the situation at the distribution center MYD represents the customer satisfaction when the vehicle arrives at each node.

**Table 9:** H-NSGA-II optimal route distribution scheme.

VN	VR	VAT	MYD
1	0–36–4-1-40–13-[51]-24–15-48-25–31–0	7:00–7:23-8:50–10:08-11:10–11:26-12:02–13:15-13:37–14:21-15:02–15:28-16:08	/-0–1-0.38124–0-1- /-0–0.65229-1–1-1- /
2	0–14–34-12-33-[52]-35–0	7:00–9:15-9:46–10:26-11:27–11:55-12:55–14:05	/-0.77831–0-1-0.32138-/-1-/
3	0–16–23-41-2-[50]-37–28–0	7:00–9:22-9:45–11:20-11:43–12:22-13:47–14:50-15:31	/-0.95967–1-1-1-/-1–1-/
4	0–8–21-3-22–0	7:00–8:34-9:04–9:35-10:13–11:31	/-1–0.36577-1–0-/
5	0–39–5-26-11-[50]-19–45-42-6–0	7:00–9:15-10:35–10:60-11:34–12:04-12:49–13:44-14:48–15:29-16:32	/-0–1-1-1-/-1–0-0-1-/
6	0–18–20-9-30–38-10-43-[49]-44–47-27-32–0	7:00–8:20-9:04–9:49-10:10–10:47-11:33–11:55-12:30–13:24-13:56–15:11-16:12–16:59	/-0.99298–0-1-0–0.9265-0–1-/-1–0-0.7016–0.78101-/
7	0–17–7-46-29–0	7:00–8:54-9:40–10:36-11:30–12:01	/-1–0.42984-0.74427–1-/

**Table 10:** NSGA-II optimal route distribution scheme.

VN	VR	VAT	MYD
1	0–31–7-2- [50] -42–45-28–0	7:00–8:02-9:50–11:38-12:18–13:03-14:13–14:54-15:35	/-0–0.986-0.959-/-0–0-1-/
2	0–44–26-19-[50]-37–32–0	7:00–9:22-11:27–11:53-12:23–13:48-14:24–15:11	/-0–1-1-/-1–0-/
3	0–16–3-41–0	7:00–9:22-10:11–10:59-12:10	/-0.960–1-0.393-/
4	0–46–47-35-[52]-6–0	7:00–9:13-9:30–11:49-12:31–14:26-15:29	/-1–0-0-/-1-/
5	0–36–40-7-29–10-43-[49]-27–0	7:00–7:23-8:13–9:27-10:29–11:27-11:49–12:24-13:54–15:18	/-0–0-0.821–0-0-1-/-1-/
6	0–23–22-25-[51]-33–48–0	7:00–9:26-10:14–11:31-11:58–13:16-14:16–15:26	/-1–0-0-/-1–1-/
7	0–8–21–0	7:00–8:34-9:04–9:52	/-1–0.366-/
8	0–18–9-30-20–38-13–0	7:00–8:20-9:13–9:34-9:52–10:59-11:38–12:10	/-0.993–1-0-0.371–1-1-/
9	0–4–14-12-15-[51]-34–24–0	7:00–8:03-9:19–10:23-11:01–11:53-13:16–14:31-15:39	/-0–0.715-1–0-/-1–1-/
10	0–1–5-11-39–0	7:00 -8:25–9:35-10:23–11:05-12:06	/-0.681–1-0-1-/

From the comparison of Figs 6 and 7, it can be seen that the distribution route with the lowest total cost obtained by the improved NSGA-II algorithm is more dense than the optimal route obtained by the improved NSGA-II algorithm. It shows that the crossing times of the optimal path represented by Fig 7 are more than those of the optimal path in Fig 6. The more path crossing times, the longer the logistics distribution path, which will lead to the increase of the number of vehicles used or the increase of the charging times of electric vehicles, and finally lead to higher vehicle use cost and charging cost.

It can also be seen from Tables 9 and 10 that the number of vehicles used in the optimal route before improvement is 10, and the minimum number of vehicles used after improvement is 7. The improved NSGA-II algorithm effectively reduces the use cost of vehicles When the traditional NSGA-II algorithm aims at the lowest total cost of logistics distribution, it only aims at the shortest path on the premise of satisfying the maximum time window of customers, so it often exceeds the expected time window of customers and leads to lower customer satisfaction The improved NSGA-II algorithm reduces the total cost of logistics distribution and improves the average customer satisfaction. It proves that the greedy algorithm and the adaptive crossover mutation strategy and mutation disturbance mechanism designed in this paper are effective. The improved NSGA-II algorithm can meet the needs of customers and allocate the service order of logistics vehicles to customers more reasonably.

Next we analysis the parameter sensitivity.

Considering the influence of road traffic congestion on the total logistics cost and average customer satisfaction of electric vehicles [29], this paper sets up two additional coefficients with traffic congestion coefficient of 1.5 and 3. 0 on the basis of an example with traffic congestion coefficient of 2.0 for parameter sensitivity analysis. From the example analysis in the previous section, it can be seen that the average value of the highest average customer satisfaction cannot detect significant differences with a high probability. When the lowest total logistics distribution cost is the goal, it can better reflect the impact of different levels of traffic congestion on electric vehicles. The impact of the vehicle logistics distribution path, therefore, select 10 times when the traffic congestion coefficient is 1.5, 2.0, 3.0, the lowest total logistics distribution cost and the operation results of the average customer satisfaction, as shown in Table 11.

**Table 11:** Solution results based on traffic congestion coefficient.

Number of runs	traffic congestion factor					
	1.5		2.0		3.0	
	Logistics Total distribution cost	Customer average Satisfaction	Logistics Total distribution cost	Customer average Satisfaction	Logistics Total distribution cost	Customer average Satisfaction
1	2651.237	0.557	3175.527	0.603	3242.579	0.585
2	2811.404	0.627	2866.601	0.595	3788.462	0.540
3	2676.168	0.516	2937.497	0.691	3116.132	0.611
4	2922.774	0.534	3067.443	0.667	3585.236	0.569
5	2659.615	0.563	3155.708	0.645	3107.732	0.617
6	2924.659	0.665	3125.728	0.584	3220.704	0.663
7	3284.934	0.579	3423.291	0.643	3230.698	0.625
8	2602.781	0.597	3269.088	0.590	3542.373	0.647
9	2984.648	0.517	3426.951	0.685	3219.635	0.567
10	2871.556	0.574	2807.701	0.667	3328.244	0.718
Near optimal solution	2602.781	0.665	2807.701	0.691	3107.732	0.718
Average	2859.838	0.573	3125.554	0.637	3338.18	0.614
Standard deviation	196.308	0.045	202.525	0.039	213.6682	0.049

From the data in Table 11, it can be seen that in the case of each degree of traffic congestion, a near-optimal solution can be found, and the near-optimal solution means the closer to the global optimum. With the increase of traffic congestion, the total cost of logistics and distribution has a clear upward trend.

When the traffic congestion coefficient is between 1.5 and 2.0, the average customer satisfaction increases with the increase of the total logistics cost, which proves the effectiveness of the algorithm in this paper The traffic congestion coefficient from 2.0 to 3.0 means that the traffic congestion becomes more serious and the total cost of logistics distribution increases while the average customer satisfaction does not show an obvious upward trend, which proves that it will become more difficult for logistics vehicles to serve within the time window expected by customers when the traffic environment becomes bad, and it will bring more severe challenges to the route planning of logistics vehicles. Therefore, it is necessary to consider the road traffic situation in the vehicle routing problem.

In addition, the standard deviation of the lowest total logistics cost (196,202, 213) and the standard deviation of average customer satisfaction (0.045, 0. 039, 0.049) under different traffic congestion coefficients are close to each other, which shows that the algorithm designed in this paper has good stability in solving the proposed problem model.

## 6 Concluding remarks

In this paper, a multi-objective vehicle routing problem with simultaneous pick-up and delivery and time window (Mo-EVRPSDPTW) is studied, and a mathematical model is established with the lowest total logistics cost and the highest average customer satisfaction. In the case of traffic congestion and dynamic power consumption, this paper designs a multi-objective genetic algorithm (NSGA-II) with fast non dominated sorting and elite reservation strategy.

Aiming at the defects of traditional NSGA-II algorithm, we design three improved strategies: using greedy algorithm and stochastic rules to construct mixed initial population, introducing adaptive crossover mutation strategy, using roulette and three neighborhood structures to enhance the disturbance mechanism of mutation operator in the stage of population crossover mutation.

Firstly, through the simulation experiments on related problems it is proved that the improved NSGA-II algorithm can effectively reduce the total cost of logistics distribution on the basis of maintaining the highest average customer satisfaction level And it can reduce the total cost of logistics distribution and improve the average satisfaction of customers when the goal is to minimize the total cost of logistics distribution.

Secondly, by discussing the route distribution scheme with the optimal total cost of logistics distribution obtained before and after the improvement of the algorithm, it is further verified that the algorithm designed in this paper can plan the vehicle service route more reasonably to meet the needs of customers when solving the MO-EVRPSDPTW problem.

Thirdly, sensitivity analysis of traffic congestion coefficient shows that different traffic environment will have an uncertain impact on average customer satisfaction and logistics cost, and further proves the effectiveness and stability of the algorithm.

In the actual road environment, different road conditions, braking and other factors will affect the power consumption of electric vehicles. Our paper do not consider non-linear charging and the possible queuing time in the charging process. Therefore, one interesting direction in our future study is to take the queuing system into account to plan the distribution route of electric vehicles. The other interesting direction is to use the idea of clustering algorithm [30–34], to improve the multi-objective evolutionary algorithm, which may reduce the chance of bad solution and improve the algorithm performance.

## Supporting information

S1 Table Customer data. (XLSX)
